# (Dis)similarities between the Decidual and Tumor Microenvironment

**DOI:** 10.3390/biomedicines10051065

**Published:** 2022-05-04

**Authors:** Jelena Krstic, Alexander Deutsch, Julia Fuchs, Martin Gauster, Tina Gorsek Sparovec, Ursula Hiden, Julian Christopher Krappinger, Gerit Moser, Katrin Pansy, Marta Szmyra, Daniela Gold, Julia Feichtinger, Berthold Huppertz

**Affiliations:** 1 Division of Cell Biology, Histology and Embryology, Gottfried Schatz Research Center for Cell Signaling, Metabolism and Aging, Medical University of Graz, Neue Stiftingtalstrasse 6, 8010 Graz, Austria; jelena.krstic@medunigraz.at (J.K.); julia.fuchs@medunigraz.at (J.F.); martin.gauster@medunigraz.at (M.G.); julian.krappinger@medunigraz.at (J.C.K.); g.moser@medunigraz.at (G.M.); berthold.huppertz@medunigraz.at (B.H.); 2 Division of Hematology, Medical University of Graz, Stiftingtalstrasse 24, 8010 Graz, Austria; alexander.deutsch@medunigraz.at (A.D.); katrin.pansy@medunigraz.at (K.P.); marta.szmyra@medunigraz.at (M.S.); 3 Division of Biophysics, Gottfried Schatz Research Center for Cell Signaling, Metabolism and Aging, Medical University of Graz, Neue Stiftingtalstrasse 6, 8010 Graz, Austria; 4 Department of Obstetrics and Gynecology, Medical University of Graz, Auenbruggerplatz 14, 8036 Graz, Austria; tina.gorsek@medunigraz.at (T.G.S.); ursula.hiden@medunigraz.at (U.H.); daniela.gold@medunigraz.at (D.G.)

**Keywords:** decidual microenvironment, tumor microenvironment, placenta, immune cells, proliferation, invasion, tumor cell, trophoblast

## Abstract

Placenta-specific trophoblast and tumor cells exhibit many common characteristics. Trophoblast cells invade maternal tissues while being tolerated by the maternal immune system. Similarly, tumor cells can invade surrounding tissues and escape the immune system. Importantly, both trophoblast and tumor cells are supported by an abetting microenvironment, which influences invasion, angiogenesis, and immune tolerance/evasion, among others. However, in contrast to tumor cells, the metabolic, proliferative, migrative, and invasive states of trophoblast cells are under tight regulatory control. In this review, we provide an overview of similarities and dissimilarities in regulatory processes that drive trophoblast and tumor cell fate, particularly focusing on the role of the abetting microenvironments.

## 1. Introduction

The placenta is a transient fetal organ and develops from fetal tissues in a complex interplay with the maternal uterine decidua, enabling unique functions such as: (1) the protection of the fetus from the immune system of the mother, (2) the anchorage of the conceptus, and (3) the provision of nutritional and gas exchange [1,2]. To accomplish this, placenta-specific trophoblast cells establish the placental barrier, promote angiogenesis, live in low-oxygen conditions, and invade maternal tissues—all while being tolerated by the maternal immune system, although fetal cells are semi-allogeneic [1]. The decidual microenvironment, in particular decidual immune cells, plays an important role in controlling trophoblast invasion and regulating the immune balance at the fetal–maternal interface [3,4]. Only a balanced activity between maternal and placental cells results in normal trophoblast invasion and successful coexistence, and a disruption of this balance could contribute to pathological conditions [3,4,5,6].

Interestingly, trophoblast and tumor cells share many striking characteristics [7,8], and both are supported by an abetting microenvironment [9]. However, how tumor cells acquire or even hijack these advantageous characteristics—which in turn contribute to tumorigenesis, tumor progression, and survival—is a matter of continuous investigation [7]. In contrast to tumor cells, the invasion of trophoblast cells into maternal tissues is precisely controlled, and the invasive behavior declines once sufficient remodeling of the uterine tissues has been achieved [4,8,10]. Therefore, the placenta has even been referred to as a “well-behaved tumor” [10], a term we discuss in more detail in the conclusion.

The study of the fetal–maternal interface in physiological and pathological conditions could facilitate the understanding of tumor biology and point towards new therapeutic routes. In cancer, the suppression of the immune response in particular has severely impaired the design of effective anti-tumor therapeutic strategies, and even the emerging immune checkpoint therapies are only effective in a small proportion of patients [11]. Hence, especially studying the interactions between trophoblasts and the decidual microenvironment could provide new perspectives (e.g., [12,13]). Here, we look beyond classic tumor biology and review similarities and differences between the cells giving life and the ones causing death, particularly focusing on their microenvironment.

### 1.1. The Placenta

The human placenta is a temporary organ of the developing embryo and fetus, that is designed to persist for about nine months once established. During this short time of existence, the placenta develops and grows while acting as the major organ of the fetus, taking over the tasks of the lungs and kidneys and serving as the main endocrine organ of the growing child [1]. The placenta is the only fetal organ that comes into direct contact with maternal blood. Hence, within the placenta, the blood streams of mother and fetus come into very close contact, only separated by a thin barrier comprising several layers of placental cells. This placental barrier is mainly made of a specific epithelial layer called the villous trophoblast, and it is the main site of exchange between both circulatory systems, allowing for the transfer of nutrients and oxygen towards the fetal side and the transfer of waste products and carbon dioxide towards the maternal side [1].

To allow maternal blood to enter the placenta, maternal vessels in the uterine wall need to be transformed and opened towards the placenta by a second trophoblast subtype, the extravillous trophoblast [14]. It was only recently shown that the invasion of extravillous trophoblasts is much less specific than thought over the last six decades [14,15]. Extravillous trophoblasts start their invasion from specific sites of proliferation (trophoblast cell columns) and reach the connective tissues of the uterus [16]. From this position, they invade all luminal structures of the uterine wall to which the placenta is attached, including uterine arteries and veins [17,18,19], uterine glands [20], and uterine lymph vessels [18,19].

Looking at pregnancy pathologies with a putative involvement of the placenta, the following pathologies and syndromes need to be listed: preeclampsia, intra-uterine growth restriction of the fetus (IUGR), and placenta accreta spectrum (PAS) disorders. Preeclampsia is most likely caused by a defect of villous trophoblast development and turnover, and it results in maternal symptoms including hypertension and the failure of kidneys, liver, and/or other major organs [21]. IUGR seems to be caused by a developmental failure of the extravillous trophoblast that results in a reduced invasion capacity and thus reduced remodeling of maternal vessels in the uterus [21]. In contrast, PAS disorders are characterized by an increased invasion capacity of extravillous trophoblasts [22].

Interestingly, a subset of preeclampsia (early-onset preeclampsia, comprising about 15% of all preeclampsia cases) is mostly associated with IUGR and thus comprises the clinically most important cases because both mother and child have increased rates of morbidity and mortality [21]. Precision medicine is already in place for this subgroup, as predictive biomarkers have been identified and are used in clinical routine today to better manage such cases and even offer first treatment options [23].

### 1.2. Decidualization

The human endometrium is a complex network of cells that undergoes a transformation process called decidualization to prepare for implantation and pregnancy [24,25,26,27]. This dynamic remodeling process transforms the endometrium into a microenvironment that is able to accommodate pregnancy and the successful coexistence of fetal and maternal cells, called the decidua [25]. In contrast to most other species, human decidualization begins in the mid-secretory phase of each menstrual cycle in response to rising levels of progesterone and estrogen and continues if a conceptus is present [24,26,28].

Decidualization includes the differentiation of endometrial stromal cells into decidual stromal cells [29]. The latter are specialized secretory cells with rounded polyploid nuclei, contain high amounts of glycogen and lipids, and synthesize a range of molecules such as components of the extracellular matrix (ECM), prolactin, insulin-like growth factor binding proteins, cytokines, and chemokines [29,30]. Decidual stromal cells are vital for the establishment of a nutritive and tolerant microenvironment for the growing placenta and affect immune tolerance, remodeling of the ECM, and angiogenesis, among others [24,25,30,31].

Decidualization is accompanied by the recruitment of immune cells, including uterine natural killer (uNK) cells, macrophages, T cells, and dendritic cells (DCs), which play key roles in immune tolerance and the promotion of pregnancy [3,32,33]. In particular, uNK cells represent a unique subtype of NK cells that exhibit weak cytotoxicity, facilitate the remodeling of spiral arteries, and promote trophoblast invasion [33,34]. Additionally, fetal extravillous trophoblasts become a vital part of the decidua when they start to invade the maternal tissues during early pregnancy [1,3,35]. Eventually, decidual stromal cells, immune cells, and extravillous trophoblasts build the interactive network required for successful pregnancy [3]. However, as pregnancy progresses, the microenvironment exhibits dynamic changes, including changes in immune cell proportions [3,36]. Indeed, defective decidua formation may contribute to infertility and certain pathological conditions during pregnancy [3,29,37].

### 1.3. Trophoblast Invasion

**Figure 1:**
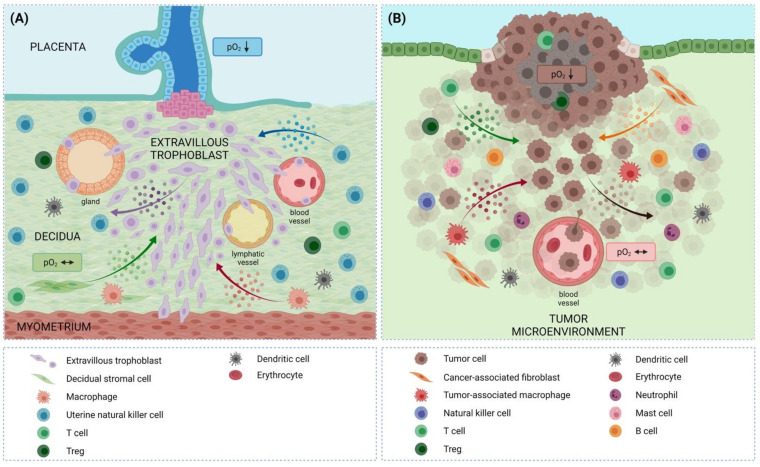
Comparison between the decidual and the tumor microenvironment. Invasive trophoblasts and tumor cells are both sustained by an abetting microenvironment. Intense crosstalk between the extravillous trophoblast/tumor cells and the cells of their microenvironment, in particular immune cells (e.g., via the secretion of molecules depicted as colored dots for certain cell types), is essential for establishing and maintaining immune tolerance/suppression towards extravillous trophoblast/tumor cells. Furthermore, cells of the microenvironment, including immune cells, are involved in regulating invasion and angiogenesis in both settings. (**A**) On the left, extravillous trophoblasts invade the uterine tissues of the mother, reaching the inner third of the myometrium. The extravillous trophoblasts invade the decidual stroma, vessels, and glands—all potential sources of nutrients. During the first trimester of pregnancy, the oxygen concentration within the villous part of the placenta has been found to be below 20 mmHg. Trophoblasts proliferate in this low-oxygen environment. From this physiologically low oxygen level (normoxia for the placenta at this stage of pregnancy), extravillous trophoblasts invade normally oxygenated uterine tissues and thus follow an oxygen gradient towards higher levels. (**B**) On the right, tumor cells can similarly invade surrounding tissues and again follow an oxygen gradient towards higher oxygen levels. Similar to extravillous trophoblasts, tumor cells proliferate in the peripheral zones around sites of low oxygen (real hypoxia), while subsequent migration and invasion take place towards higher oxygen levels. Created with BioRender.com (accessed on 23 April 2022).

Trophoblast invasion starts as early as the fetal blastocyst implants into the maternal uterus. Early attachment and implantation (i.e., invasion) into the maternal decidua begin with the outer trophoblast layer of the blastocyst, called the trophectoderm, and are completed around day 11 post conception (p.c.). The first differentiation of those trophectoderm cells in direct contact with uterine epithelial cells results in the formation of the multinucleated syncytiotrophoblast, which is an invasive tissue of the embryo at this time. The further differentiation of the trophoblast layers takes place rapidly. Within the first weeks of pregnancy (day 14 p.c.), mononucleated cells (cytotrophoblasts) differentiate from the trophectoderm, detach as extravillous trophoblasts from the developing placenta, and continue invading the maternal decidua, finally invading all potential sources of nutrients (the decidual stroma, vessels, and glands) (Figure 1A) [1,35].

Invading extravillous trophoblasts fulfil two main functions during pregnancy: (1) attaching the placenta to the uterus and (2) connecting the placenta with maternal tissues, vessels, and glands for the exchange of nutrients and the drainage of waste/debris [17,38]. A fundamental characteristic of extravillous trophoblasts is that they stop proliferating as soon as they begin to invade [1,39].

Factors secreted by extravillous trophoblasts may contribute to controlling their invasion in an autocrine manner, as well as influence immune cell function within the decidua in a paracrine way [40,41]. Probable ligand–receptor interactions between trophoblasts and all other cell types within the decidua (such as immune cells, decidual stromal cells, endothelial cells, perivascular cells, and glandular epithelial cells) were investigated in a recent single cell RNA-seq study [9].

The influence of numerous factors on trophoblast invasion has been investigated. In addition to various proteases secreted by extravillous trophoblasts (such as the matrix metalloproteinases (MMPs) MMP-2, MMP-3, and MMP-9), MMP-15 was also recently discovered as a crucial factor for trophoblast invasion. Within the placenta, MMP-15 is restricted to the invasive extravillous trophoblast, and its in vitro silencing leads to restricted trophoblast outgrowth, though it has demonstrated no influence on proliferation or apoptosis [42,43]. Another important group of proteins relevant for trophoblast invasion is the integrin family. Dependent on the composition of the various subunits, some integrins promote adhesion while others facilitate invasion [44,45]. MMPs and integrins are key players in both trophoblast and cancer invasion. Examples of MMPs playing significant roles in both settings—trophoblast and tumor invasion—are MMP-2 and MMP-9, both known to be important facilitators of invasion [46,47,48,49]. In contrast, MMP-15 has no influence on trophoblast apoptosis but does inhibit apoptosis in several tumor cell lines [50].

### 1.4. Tumor Microenvironment

Cancers are a complex and heterogeneous group of diseases that affect millions of people. Yet, tumor cells exhibit certain advantageous characteristics called the hallmarks of cancer, including growth and proliferative advantages, modified response to stress, metabolic reprogramming, invasion and metastasis, stimulated angiogenesis, immune evasion, and an abetting tumor microenvironment (TME) [51,52]. The TME is a complex network of non-malignant stromal cells (including fibroblasts and endothelial cells), immune cells, vessels, nerve fibers, ECM proteins, and secreted signaling molecules within and around the tumor (Figure 1B) [53,54,55].

Tumor cells can sculpt the TME to support tumor survival, progression/metastasis, and drug resistance [56,57]. Intercellular communication between the TME and tumor cells is highly complicated and dynamic, occurring via direct cell-to-cell interactions, such as membrane-tethered receptor–ligand binding or signaling through gap junctions and tunneling nanotubes, and via indirect mechanisms, such as the secretion of cytokines, chemokines, growth factors, exosomes, and metabolites [57,58]. Generally, the TME is a highly complex ecosystem that exhibits profound heterogeneity within tumors, between different malignancies, and between individual patients and that crucially affects tumor biology [55,59,60,61,62,63,64]. The TME may be envisioned as a combination of specialized niches that can overlap as well as communicate, including the hypoxic, immune, acidic, innervated, metabolic, and mechanical niches [55]. Advancing the understanding of the TME and its crosstalk with tumor cells could promote the development of new and tailored therapeutic regimens [55,57,65]. In particular, considering the profound complexity and heterogeneity of tumors and their microenvironments, combination therapies hold promise [11,53,57].

## 2. Tumor and Decidual Microenvironment: How Much Do They Have in Common?

Trophoblast and tumor cells are both supported by an abetting microenvironment (Figure 1) that influences/regulates invasion, angiogenesis, and immune tolerance/evasion, among others. In both settings, the cells invade from low to high oxygen levels, show very close interactions with vessels on their way through the tissue, and are tolerated by/evade the immune system. This is facilitated by the intense crosstalk of the extravillous trophoblast/tumor cells with the cells in their microenvironment (Figure 1). In the subsequent sections, we offer an overview of similarities and dissimilarities in regulatory processes driving trophoblast and tumor cell fate, with an emphasis on the role of the abetting microenvironments.

### 2.1. Growth Suppression in Tumor and Decidual Microenvironment

One biological capability acquired during the multistep development of human tumors is the hallmark of evading growth suppressors, representing the competence of cancer cells to circumvent powerful programs that negatively regulate cell proliferation [52]. Many of these programs depend on the actions of dozens of previously identified tumor-suppressor genes. Amongst these, many have been characterized as bona fide tumor suppressors by gain- or loss-of-function experiments in animal models, including the classical tumor suppressors retinoblastoma (Rb)-associated proteins, tumor protein p53, and phosphatase and tensin homolog (PTEN). While some of these factors transduce growth-inhibitory signals that largely originate outside the cell, others receive inputs from intracellular operating systems based on stress and abnormality sensors inside the cell. Extrinsic signals may originate from fibroblasts in the TME. In this environment, it seems as if such signals help cancer cells to evade various forms of growth suppression. Co-culture experiments have clearly shown that normal connective tissue fibroblasts, but not cancer-associated fibroblasts, can inhibit the growth of cancer cells in a mechanism that requires the contact of fibroblasts with cancer cells [66]. Thus, normal fibroblasts may serve as extrinsic growth suppressors.

As mentioned above, endometrial stromal cells in women of childbearing age are subject to cyclic decidualization, including the transformation of these cells into secretory decidual stromal cells. Their secretion products have a variety of functions including the control of trophoblast invasion. Culture of differentiating trophoblasts with decidual stromal cell-derived culture supernatant was shown to induce the phosphorylation of Smad2/3 [67], suggesting signaling through members of the transforming growth factor (TGF)-β superfamily. TGF-β signaling is one of the most extensively studied tumor-suppressor pathways in epithelial cell malignancies. In fact, most epithelial cells are growth-inhibited by TGF-β, and the loss of this response has been suggested as a key event in the progress towards malignancy [68]. Recent studies in normal epithelial cells showed that TGF-β1 induced the expression of growth suppressor p12, which in turn inhibited growth via the CDK2-catalyzed phosphorylation of Rb [69]. In contrast, deficiency in p12 expression resulted in partial resistance to TGF-β1-mediated inhibition of cell proliferation. The molecular mechanisms driving trophoblast invasiveness are considered to be identical to those of cancer cells, even though their proliferation, migration, and invasiveness in situ are stringently controlled by decidua-derived TGF-β [70]. In contrast to normal extravillous trophoblasts, hyperproliferative and hyperinvasive premalignant trophoblasts, as well as malignant trophoblast-derived choriocarcinoma cell lines such as JAR and JEG-3, have been shown to be TGF-β-resistant. Notably, the loss of TGF-β response in malignant trophoblasts was explained by the loss of expression of the *SMAD3* gene. Moreover, differential mRNA display of normal and premalignant trophoblasts revealed deregulation of numerous genes in premalignant trophoblasts, with potential oncogenic and/or tumor-suppressor functions. Amongst these, the loss of insulin-like growth factor binding protein 5 (IGFBP5) and insulin-like growth factor 2 receptor (IGF2R) was suggested to enable unrestricted proliferation in an IGF-1-rich microenvironment of the fetal–maternal interface [70,71].

However, whether decidual stromal cell-derived factors, such as TGF-β superfamily members, can directly affect the classical tumor suppressors Rb, p53, and PTEN in human trophoblasts has not yet been described in great detail. Most knowledge has been gained from different mouse models, substantiating the fundamental role of tumor-suppressor genes during placental development in mice. Initial morphological surveys suggested that the deletion of Rb leads to extensive microanatomical changes in the mouse placenta, including reductions in the total volume and vasculature of the placental labyrinth, increased infiltration from the spongiotrophoblast layer to the labyrinth layer, and clustering of labyrinthic trophoblast [72]. For the human placenta, immunohistochemistry revealed that the retinoblastoma family members, p107 and Rb2/p130, are most abundantly expressed during the first trimester of gestation and progressively decline to barely detectable levels in the placenta in late gestation [73].

In addition to Rb, a growing body of evidence suggests that p53 plays fundamental roles in placental development and physiology. Placental tissue from p53−/− mice at E14.5 have shown structural abnormalities, including mild-to-moderate labyrinth trophoblast hyperplasia, collapsed vasculature, and nuclear enlargement of labyrinthic trophoblast (polyploidy) [74]. In general, the stabilization of p53 inhibits cell proliferation through the activation of its transcriptional target p21, which in concert with p16 maintains the Rb protein in its hypophosphorylated and active state. An active Rb protein suppresses the transcription factor E2F1-dependent expression of genes that regulate the progression of the G1/S phase of the cell cycle, thereby irreversibly blocking cell cycle entry [75,76]. The consequence of this process is cellular senescence, a state of irreversible, terminal arrest of cell proliferation. A recent proteomics approach identified candidate proteins involved in p53 high-molecular-weight complex formation that were suggested to be responsible for the inactivation and stabilization of p53 in primary first trimester trophoblasts. Amongst the binding partners, glucose-regulated protein 78 (GRP78) was demonstrated to be involved in p53 stabilization and trophoblastic invasion since the decreased expression of membrane GRP78 decreased p53 stability and increased the invasion of trophoblasts [77]. p53 is a transcription factor that can upregulate MMP-2 and downregulate MMP-1, -9, and -13. Thus, the sequestration of p53 by GRP78 or many other binding partners may affect the invasiveness of cells. In contrast to the observations in trophoblasts, metastatic cells show increased levels of GRP78, which has been suggested to promote tumor metastasis through the binding of α2-macroglobulin to GPR78 at the cell surface, thereby activating the PAK2 pathway [78]. Whether GRP78 binds to p53 and thereby affects MMP activity in tumor cells has not been described so far. Recently, the E3 ligase TRIM72 was shown to directly interact with p53 and promote its ubiquitination and proteasomal degradation, leading to reduced apoptosis and enhanced migration in trophoblasts [79]. In addition to ubiquitination, a complex array of post-translational modifications, including phosphorylation, sumoylation, neddylation, acetylation, and methylation, affects the stabilization of p53 or its sequestration by many binding partners to modulate p53 activity (comprehensively summarized in [80]). Hence, a comparison of the post-translational modifications of p53 and its stability between trophoblasts and metastatic cells represents an intriguing issue that could further contribute to our understanding of differences between placentation and cancer.

PTEN, another tumor suppressor that has attracted increasing attention in tumor research during the last two decades, plays a pivotal role in apoptosis, cell cycle arrest, and possibly cell migration [81]. PTEN functions through converting phosphatidylinositol triphosphate into phosphatidylinositol 4,5-bisphosphate, thereby negatively regulating the Akt/PKB signaling pathway [82]. In normal human pregnancy, placental PTEN expression decreases with progressing pregnancy and placental development [83]. Endometrial PTEN expression is higher during the first trimester of pregnancy compared to any time in the normal menstrual cycle, and it is directly regulated by the ovarian steroids, estradiol and progesterone. While estradiol is suggested to downregulate PTEN activity by increasing its phosphorylation, progesterone is likely to regulate the PTEN pool by decreasing its phosphorylation and increasing its protein level [84]. Decidual PTEN expression is significantly increased in cases of spontaneous abortion compared to controls [83]. Recent in vitro studies with the trophoblast cell line HTR-8/SVneo suggested that PTEN is involved in the regulation of trophoblast invasion [85]. Moreover, PTEN is part of the hypoxia-responsive network in the placenta, including HIF1α and microRNA-20a as upstream regulators, and it has been shown to be upregulated in trophoblast-derived choriocarcinoma JAR cells cultured under 2% oxygen for 24 h [86,87].

Overall, data from mouse models and in vitro experiments suggest an important role for tumor-suppressor genes in regulating trophoblast cell expansion and invasion processes. While a high level of cell proliferation is required for the rapid growth of embryonic and placental tissues in the early stages of pregnancy, transition to cellular differentiation and senescence is mandatory towards term. The disruption of this balanced regulation manifests in the pathogenesis of gestational trophoblastic disease, characterized by abnormally proliferating trophoblastic tissues, including partial and complete hydatidiform moles, invasive moles, choriocarcinoma, and placental-site trophoblastic tumors [88].

### 2.2. Proliferative Signaling in Tumor and Decidual Microenvironment

During placental development, trophoblasts form clusters of highly proliferative cells at the attachment site to the uterine wall (trophoblast cell columns), where they proliferate for a limited time period [35]. At around mid-gestation, the pool of proliferative cells seems to be mostly exhausted. All the non-proliferative daughter cells of this pool undergo differentiation into extravillous trophoblasts and acquire an invasive phenotype (Figure 1) [1,35]. At the sites of the trophoblast cell columns and in the course of the rapid proliferation, trophoblasts engage in common proliferative signaling pathways to sustain their growth in a tightly regulated manner, by which they are intrinsically and extrinsically instructed to proliferate [89]. As in other healthy tissues, trophoblasts need a mitogenic growth signal to initiate division [90]. The most notable growth factors guiding trophoblast proliferation are epidermal growth factor (EGF), hepatocyte growth factor (HGF), IGF, vascular endothelial growth factor (VEGF), placental growth factor (PlGF), and TGF [91,92]. Most of the corresponding receptors are receptor tyrosine kinases (RTK), which activate downstream pathways such as Ras/Raf/MAPK or PI3K/AKT, resulting in cell division [89]. Importantly, the RTKs in trophoblast are activated after receptor dimerization and ligand binding, thereby causing the phosphorylation of the receptor C-terminal tail [91]. Some of the proteins participating in proliferation signaling are encoded by proto-oncogenes (e.g., *RAS*), which are expressed at similar levels in transformed tumor cells [89]. In the placenta, however, these proto-oncogenes are expressed with a cell-type- and time-dependent specificity, confirming the high level of thoroughly regulated proliferation [89,93].

The constituents of the decidual microenvironment participate in placentation and trophoblast proliferation and invasion. Many growth factors and cytokines, such as EGF, TGF-β, and tumor necrosis factor alpha (TNF-α), that are secreted by decidual stromal cells and uNK cells regulate trophoblast function in a paracrine manner [24,94]. These factors may also be secreted by the trophoblast and act in an autocrine manner [89] (Figure 1). In the context of cell proliferation, uNK cells produce signaling molecules such as cytokines (e.g., TNF-α), growth factors (e.g., TGF-β), angiogenic factors (e.g., VEGF and PlGF), and MMPs, all of which contribute to the regulation of the proliferative capacity of trophoblasts [94]. Through MMP activation, the growth factors embedded in the ECM can be released and activated [95].

Even though trophoblasts and transformed tumor cells share similar molecular circuitries regulating proliferation, there are major differences in the regulatory pathways. Tumor cells hijack the proliferative signaling to sustain their unlimited growth [52], thus avoiding the spatiotemporal regulation present in placental development [91]. In tumor cells, the sustained proliferation is mostly intrinsically regulated by underlying mutations in proto-oncogenes that encode members of the proliferative signaling pathways [52,96,97]. For example, gain-of-function mutations in the various subdomains of an RTK lead to the constitutive activation of the RTK, typically in the absence of a ligand. Additionally, the overexpression of RTKs, usually arising from the genomic amplification of the *RTK* gene, leads to increased local concentrations of receptors [98,99,100]. Hence, tumor cells uncouple their proliferative signaling from the extracellular proliferation instructions. Still, tumor cells rely on their microenvironment to sustain their growth. Similar to the decidual microenvironment, proteolytic enzymes are a major component of the TME, enabling the release and activation of growth factors [101]. Tumor cells are also capable of modifying their microenvironment, either by secreting factors or by direct cell–cell contact with the purpose of attaining nutrients, proliferative stimuli, and immune evasion [53].

The knowledge coming from placental research can be used to identify the regulatory modalities that could be used for cancer treatment. Interestingly, many targeted therapeutics already target a variety of proteins with different functions shared by trophoblast and cancer cells (such as growth pathway signaling molecules or enzymes regulating invasion (reviewed in [102])). A recent study suggested that the permissiveness of placental stroma to trophoblast invasion in mammals correlates with higher susceptibility to malignancy [103]. Although based on in vitro models that compared the permissiveness of human and bovine fibroblasts to trophoblast invasion, this study illustrated the importance of the microenvironment for tumor growth, proliferation, and invasion, which could be traced back to the placenta’s distinct invasive properties in mammals. However, this hypothesis is still under investigation [104].

### 2.3. Angiogenesis in Tumor and Decidual Microenvironment

The growth and development of a tissue, an organ, or an organism require an adequately formed vasculature to ensure oxygen and nutrient supply. Angiogenesis is the formation of new blood vessels from a pre-existing vasculature. It is a multistage process tightly regulated by pro- and anti-angiogenic factors. In adults, angiogenesis is a rare phenomenon, and endothelial cells remain mostly quiescent. Thus, for the induction of angiogenesis, endothelial cells require activation by pro-angiogenic signals. Once activated, endothelial cells produce proteases, detach from the endothelial monolayer, and migrate towards the concentration gradient of the pro-angiogenic signals. VEGF, PlGF, and basic fibroblast growth factor (FGF2) are among the most potent pro-angiogenic factors, but the list of angiogenic stimuli covers a plethora of growth factors, cytokines, and hormones [105,106].

To supply the developing and growing fetus with sufficient nutrients and oxygen, the decidual vasculature enlarges and adapts with angiogenesis and vascular growth, starting with implantation. Impaired decidual angiogenesis is implicated in implantation defects and early pregnancy loss [107]. Trophoblasts possess a unique endocrine ability and are a source of hormones, growth factors, and cytokines with pro-angiogenic effects [108,109,110] (Figure 1). These factors promote decidual angiogenesis in the placental bed; a culture medium conditioned by blastocysts was found to stimulate endometrial angiogenesis in vitro [111], suggesting that the trophectoderm of the early embryo already releases pro-angiogenic signals. During early pregnancy, trophoblasts differentiate to the syncytiotrophoblast, the epithelial cover of the placental villous trees. As such, the syncytiotrophoblast secretes human chorionic gonadotropin to maintain pregnancy, stimulating angiogenesis and recruiting pericytes [112]. Indeed, the angiogenesis of decidual blood vessels predominantly occurs around the implantation site [113]. However, decidual angiogenesis is a physiological, controlled, and limited process. Therefore, not only is the microenvironment of the decidua enriched in pro-angiogenic signals but trophoblasts also produce molecules that inhibit angiogenesis [114,115], highlighting the need for the spatial restriction of vascular sprout outgrowth.

The establishment of a surrounding vasculature significantly promotes the development and growth of a tumor. The connection to the blood system enables oxygen and nutrient supply, as well as spreading of the tumor via the bloodstream (Figure 1). Thus, the microvessel density of a tumor is associated with increased tumor growth and metastasis [116]. Additionally, tumor cells have the ability to secrete growth factors and pro-angiogenic signals that can stimulate the ingrowth of blood vessels [117]. Hypoxia arising within the growing tumor may further enhance the expression and release of pro-angiogenic factors including VEGF, angiopoietins, and FGF2 [118,119]. Trophoblasts also respond to low oxygen with the increased expression of VEGF [120], but due to the particular paracrine activity of trophoblasts and the release of a variety of different pro-angiogenic factors that are not regulated by low oxygen, the role of low oxygen in decidual angiogenesis may not be as important as in solid tumors.

Apart from the promotion of decidual angiogenesis, trophoblasts can further increase the blood supply to the placenta by remodeling the uterine vasculature. Invading trophoblasts change phenotype [121], replace the endothelium of the uterine spiral arteries, and convert them into large, low-resistance vessels [122]. Vasculogenic mimicry, i.e., the formation of microvascular channels by non-endothelial cells, also exists in some kinds of aggressive tumors; tumor cells form vessels to increase nutrient and oxygen supply [123]. Interestingly, extravillous trophoblasts and tumor cells invade lymph vessels and reach local lymph nodes, again showing similarities in their invasive behavior [18].

Leukocytes play a central role in angiogenesis (Figure 1). Upon recruitment, leukocytes extravasate from the circulation into their target tissue, where they differentiate and produce and release large amounts of cytokines and growth factors that modulate the microenvironment and may act in a pro-angiogenic manner [124,125]. Trophoblasts recruit different types of leukocytes into the decidua, including uNK cells [126,127,128,129], decidual macrophages [127,130,131], and DCs [126,132]. All of them secrete a plethora of pro-angiogenic factors. DCs are thought to contribute to decidual angiogenesis during embryo implantation [133]. Later in pregnancy, uNK cells and macrophages are thought to facilitate spiral artery and tissue remodeling by extravillous trophoblasts [134,135]. Additionally, tumor angiogenesis involves various classes of leukocytes [136,137], which are recruited by tumor cell-derived chemokines [138,139]. The most abundant leukocytes present in tumors are tumor-associated macrophages. In fact, the pro-angiogenic action of tumor-associated macrophages has been shown in many types of cancer [140,141,142]. Moreover, NK cells, alone or in combination with mast cells [143], and DCs [142] have been shown to promote tumor angiogenesis.

### 2.4. Evasion of Immune Destruction in Tumor and Decidual Microenvironment

In addition to fetal trophoblasts and maternal decidual stroma cells, the decidua contains maternal immune cells, including uNK cells, macrophages, T cells, and DCs, though B cells are rarely found (Figure 1). Interestingly, maternal immune cells account for 30–40% of all decidual cells in early pregnancy [127,144]. All different immune cell types and the stromal cells are involved in promoting immune cell homeostasis during pregnancy [145]. In the following section, the different decidual immune cells and their functions are briefly summarized, with a focus on their immunosuppressive properties.

uNK cells are the most important immune cells during pregnancy, comprising the largest immune cell population in the decidua (50–70% of all decidual immune cells) [146]. They possess a CD16^neg^ CD56^high^ surface phenotype. This phenotype is similar to that of blood-derived cytokine-producing NK cells, which possess a weak cytotoxicity and produce a variety of cytokines [127,147,148]. uNK cells also produce cytokines such as IL-8 and IL-10 to regulate trophoblast invasion and VEGF-C, Arg1, Arg2, and TGF-β to initiate artery remodeling within the decidua [134,149]. In addition, uNK cells can produce G-SCF, GM-SCF, m-SCF, and TNFα, which are involved in achieving successful pregnancy [150]. The cytotoxicity of uNK cells is regulated by the HLA class I molecules expressed by extravillous trophoblasts and the activating or inhibitory receptors on the uNK cells [150]. Extravillous trophoblasts express HLA-C, HLA-E, and HLA-G but not HLA-A and HLA-B. This HLA expression pattern is different to that of most somatic cells [151]. uNK cells express inhibitory receptors, including LILRB1, KIR2DL4, and CD94/NKG2A [144], and their cytotoxic effects are suppressed once an interaction occurs between these inhibitory receptors and the ligands—namely HLA-C, HLA-E, and HLA-G—expressed by the trophoblasts [147]. uNK cells also express activating receptors, NKp46, NKp44, NKp30, NKG2D, and CD94/NKG2C [152], but the cytolytic effects mediated by these receptors are inhibited by macrophages through a TGF-β1-dependent mechanism [148].

Decidual macrophages are the second largest category of immune cells, accounting for approximately 20% of all decidual immune cells [153,154]. They can be divided into CD209+ and CD209− subgroups by flow cytometric analysis. The CD209+ macrophages may identify pathogens in the decidua and are therefore implicated in immune defense against pathogens. In contrast, CD209− macrophages tend to have a similar phenotype to M2 macrophages and are implicated in immunosuppressive processes via the secretion of IL-10 [155,156].

Decidual T cells account for 10–20% of decidual immune cells and mainly consist of CD3+ T cells, whereas γδ−, CD3+, CD4−, CD8−, and NK-T cells are rarely found. CD3+ T cells can be further subclassified as CD4+ (also called T helper cells (Th), accounting for 30–45% of T cells) and CD8+ T cells (also called cytotoxic T cells, accounting for 45–75% of T cells). Among the T helper cells, FOX3p+ regulatory T cells (Tregs), Th2 cells, Th17 cells, and Th1 cells account for 5%, 5%, 2%, and 5–30% of decidual T cells, respectively [157]. Decidual maternal cytotoxic T cells have the potential to recognize semi-allogeneic fetal cells directly via HLA class I molecules on extravillous trophoblasts or indirectly via maternal antigen presenting cells [158]. However, it was shown that compared to peripheral cytotoxic T cells, cytotoxic T cells in the decidua express higher co-inhibitory receptors, such as PD1 and TIM3, which bind to their corresponding ligands, such as PD-L1 and PD-L2, expressed on extravillous trophoblasts. This co-inhibitory receptor–ligand interaction results in the induction of immune tolerance [159]. Decidual Th cells, mainly Th1 and Th2 cells, also possess the ability to recognize fetal cells [160]. When activated, Th1 and Th2 cells produce inflammatory cytokines to promote inflammatory processes. It was shown that Th2 cells in the decidua possess a different differentiation state, causing Th1-repressing properties that are mainly IL-10-mediated, than Th2 cells in any other type of tissue [161,162]. Tregs are known to maintain immune homeostasis by suppressing the activity of other immune cells after the appropriate elimination of the invading organisms [163]. The mechanism by which Tregs mediate immunologic tolerance in the decidua is not yet fully understood. It was shown that Tregs stimulate decidual stromal cells, macrophages, and DCs to express indoleamine 2,3 dioxygenase (IDO), whose metabolite is toxic to T cells [164,165,166].

Decidual DCs are less abundant but possess the ability to present fetal antigens [167]. These cells have been poorly investigated in the decidua so far, but they possess an important role in regulating the activated decidual T cell function [168,169]. Two different DC phenotypes exist in the human decidua: a large number of immature DC-SIGN+ DCs and a small number of CD83+ DCs [33]. Physiologically, CD83+ DCs possess the ability to migrate to the secondary lymphoid organs to initiate T cell activation, whereas DC-SIGN+ DCs promote Th cell responses and are implicated in the recruitment of uNK cells [168,170]. In the absence of stimulation or in anti-inflammatory conditions, DCs differentiate into tolerogenic DCs and produce anti-inflammatory cytokines [171].

**Figure 2:**
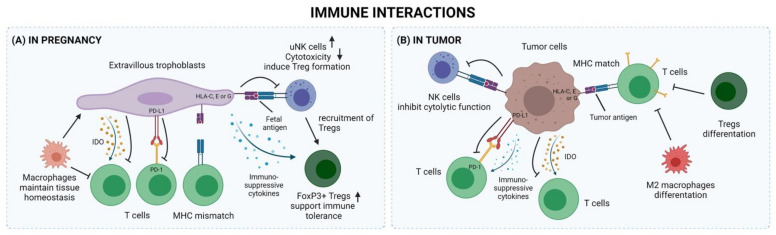
Comparison of the immune interactions found in (**A**) the decidual microenvironment and (**B**) the tumor microenvironment. In both, similar immune cells (NK cells, T cells, Tregs, and macrophages) are found. Both may also use similar mechanisms of immune evasion to suppress the immune responses of the host, mediated by the secretion of immunosuppressive cytokines, metabolites (e.g., IDO), co-inhibitory signals, non-classical MHC class I molecules and/or by the recruitment of Tregs and/or NK cells. Secreted factors are shown as dots. Created with BioRender.com (accessed on 23 April 2022).

The immune cells of the TME have been intensively studied during the last few years [172], and similarities to those of the decidual microenvironment have been observed (Figure 2). For example, it has been suggested that NK cells in the TME could exhibit uNK-cell-like features, have low-cytotoxic capacity, and promote angiogenesis [173,174,175,176,177,178,179].

Both the decidua and TME contain NK cells, macrophages, T cells, Tregs, and DCs [180,181,182,183,184]. Additionally, B cells and neutrophils are also frequently found in the TME [180,181,182,183] but rarely found in the decidua. Tumor cells also express HLA-C and the non-classical MHC class I molecules HLA-E and HLA-G similarly to extravillous trophoblasts, resulting in NK and cytotoxic T cell inhibition in both conditions [185,186,187,188,189,190,191]. In tumors, immune cells can be activated by neoantigens, which are expressed by acquired genetic alterations in the malignant cells. In the decidua, extravillous trophoblasts express fetal antigens due to their semi-allogeneic background. Hence, extravillous trophoblasts in the decidua and malignant tumor cells may use similar immune inhibitory mechanisms to suppress the immune response of the host. In tumors, this is achieved by expressing immune inhibitory ligands, such as PD-L1, PD-L2, CD80, CD86, and TIM3L, on the tumor cell surface. These inhibitory ligands interact with the corresponding co-inhibitor receptors, such as PD1, CTLA-4, and TIM3, on immune cells, therefore inhibiting the anti-tumor immune responses [182,184,192]. In addition, anti-tumoral immune responses are suppressed by the expression of IDO in the malignant cells or by other immune cells such as Tregs and M2 macrophages [193,194,195,196,197,198,199,200].

### 2.5. Promotion of Invasion in Tumor and Decidual Microenvironment

With their capability to invade, proliferate, and induce a blood supply, cancer cells show substantial similarities but also dissimilarities to extravillous trophoblasts in early pregnancy, when they invade the uterine decidua [102,103]. In both settings, conditions for successful cellular invasion include alterations in cellular programs responsible for cell adhesion, protease secretion, and the presence of growth factors, resulting e.g., in reduced cell-to-cell contacts [102,201,202]. During both processes, high amounts of MMPs are produced and secreted, thus causing the degradation of ECM components [4,102,203,204,205,206].

While cancer cells spread throughout the invaded tissues, even forming distant metastases, extravillous trophoblasts follow a highly organized differentiation pattern from proliferation to invasion [102,207]. This organized pattern is regulated by different signaling pathways. The Janus kinase-signal transducer and activator of transcription (JAK/STAT) pathway is suggested as one of the most significant signaling pathways in this context [208]. In many cell types, a plethora of cytokines and growth factors activates the JAK/STAT pathway, leading to the phosphorylation of the cytoplasmic STAT3. STAT3 then translocates into the nucleus and binds and activates the expression of its target genes (mainly MMPs) involved in invasion [209,210,211,212]. In addition, STAT3 signaling also influences the expression and function of a number of genes that are crucial for cell survival, cell proliferation, angiogenesis, and immune evasion. Regarding cancer cell invasion, the activation of STAT3 by oncogenic proteins is one of the most common pathways, resembling the signaling routes of trophoblast invasion [210]. More precisely, it was found that epidermal growth factor (EGF) is associated with the invasiveness of tumors and is also responsible for stimulating the motility of extravillous trophoblasts [102,213,214].

The decidua plays an active role in regulating the process of trophoblast invasion rather than just being a stiff matrix that passively waits to be invaded [35,208,215]. Decidual stromal cells express and secrete a variety of different cytokines and growth factors, including LIF, IL-6, IL-11, IL-15, CXCL-10, HGF, and GM-CSF, which are known to be direct regulators of trophoblast invasion (Figure 1) [24,208,209,216]. These factors are able to induce the chemotaxis of invasive trophoblasts and guide them towards uterine vessels, especially uterine spiral arteries [216,217]. Decidual IL-6 upregulates the expression of MMP-2 and MMP-9 in extravillous trophoblasts [208], which is counteracted by tissue inhibitors of metalloproteinases (TIMPs), also secreted by decidual stromal cells [4,203,205]. This way, the decidua can actively promote and limit trophoblast invasion. MMPs also play important roles in cancer invasion. In particular, MMP-2 and MMP-9 are key factors in this process [50].

Interestingly, as described for trophoblast invasion, IL-6 is also expressed and secreted by cancer cells and cells in the TME. Thus, the IL-6/JAK/STAT-3 pathway appears to regulate a majority of invasion-promoting functions in various cancer types [218,219,220], as well as in trophoblasts. In addition, chemokines are known to be frequently expressed in the inflammatory microenvironment of tumor cells. Ren et al. showed that the overexpression of CXCL10 significantly enhances the migration, invasion, and metastasis of hepatocellular carcinoma, thereby playing an important role in the regulation of cancer invasiveness [221]. Recent findings by Godbole et al. described an additional regulatory pathway for trophoblast invasion based on a two-step process controlled by decidual cells. The downregulation of the homeobox transcription factor HOXA10 in decidual cells leads to a burst in the production of LIF and IL-6, which activates STAT3 in trophoblasts and stimulates the expression of MMPs in a paracrine manner. Decidual cells containing HOXA10 exhibit limited production of pro-invasive molecules, thus inhibiting invasion. It is assumed that the activity of HOXA10 in vivo prevents premature invasion and helps define the limit of invasive depth within the decidua [209]. According to recent studies, HOXA10 also plays a role in the development of a variety of cancer types. The disruption or abnormal expression of HOXA10 thereby promotes the malignant behavior of tumor cells [222,223], especially in prostate cancer, in which a tumor-suppressor role of HOXA10 was identified [222]. Furthermore, investigations using various trophoblast cell models have demonstrated that transcription factors, including GCM1, AP2α, and FOS-like 1, are also involved in the control of trophoblast-specific gene expression and thus invasion [35,224,225,226].

In addition to decidual stromal cells, there is evidence that the invasion of extravillous trophoblasts is influenced by immune cells [41,227] (Figure 1). In utero, high numbers of maternal immune cells can be detected prior to the implantation of the embryo, and thus may be involved in preparing the uterus for pregnancy [227]. uNK cells and macrophages, especially, are found in proximity to extravillous trophoblast cells, promoting invasion and tissue remodeling [228,229]. In vitro and animal model studies have suggested that these immune cells promote decidual and spiral artery invasion through the secretion of cytokines, angiogenic mediators, and growth factors [228,229]. In the tumor setting, tumor-associated macrophages have been linked to tumor progression and metastasis [230,231]. In contrast, increased intratumoral levels of activated NK cells appear to prevent cancer invasion and thus metastasis [231,232]. However, a number of evading strategies have been observed in tumors, including limited NK cell infiltration into solid tumors and NK cell dysfunction [172,185,232,233]. A number of reports have even suggested that NK cells could adopt a uNK cell-like phenotype [173,174,175,176,177,178,179].

Furthermore, the invasion of trophoblast cells also seems to be hormonally regulated. During implantation, the endometrium forms the decidua in response to release of the ovarian hormone progesterone (P4). Decidualized stromal cells are characterized by secreting prolactin, which stimulates trophoblast invasion and the formation of ciliated glandular epithelial cells in vitro [215,234]. The secreted factors coming from the uterine glands regulate the development and function of the placenta, including trophoblast attachment, invasion, and growth [234,235]. Whether the secretions of the uterine glands directly stimulate trophoblast invasion remains elusive. Increased prolactin levels have also been linked to the higher invasiveness of specific tumor types [234,236]. This correlation seems to be particularly strong in breast cancer. The similarity between prolactin and growth hormones and its influence on the JAK/STAT signaling pathway strongly indicate its impact on tumor invasiveness [236]. Hence, it is assumed that both trophoblast and tumor invasion are also affected and regulated by the hormonal products produced by cells of their microenvironments [215,236].

### 2.6. Chemo-Physical Aspects of Tumor and Decidual Microenvironment: Oxygen as an Example

The chemo-physical aspects of the TME compared to the microenvironment during trophoblast invasion appear to be similar in a number of ways, especially when it comes to oxygenation. Oxygen levels have been shown to be low in both tumors and first trimester placenta. In addition, both scenarios show high proliferation at sites of low oxygen and migration and invasion towards higher levels of oxygen (Figure 1).

Maternal blood flow into a first trimester placenta is blocked by plugs of invaded endoarterial trophoblasts and thus, only a plasma flow through the placenta is established at this time of pregnancy [237]. This allows only physically solved oxygen to enter the placenta, resulting in pO_2_ levels between 10 and 25 mmHg [238]. As the sources for invading trophoblasts and sites of highest trophoblast proliferation, the trophoblast cell columns are bathed in this plasma flow and hence develop in a low-oxygen environment [239,240]. It needs to be stressed here that in a first trimester placenta, the low pO_2_ levels are normoxic and there are no such low oxygen levels in the surrounding maternal tissues [239]. Starting from the cell columns, trophoblasts start to differentiate and invade the uterine wall tissues with much higher pO_2_ levels (from 55 to 70 mmHg [238]) than those present in the placenta. Hence, there is migration along an oxygen gradient from a low-oxygen environment with proliferation to a high-oxygen environment with invasion [239], similar to tumor cells escaping from the tumor mass and invading the surrounding tissues.

With the onset of maternal blood flow through the placenta at the beginning of the second trimester of pregnancy, the pO_2_ levels within the placenta rise to values between 40 and 55 mmHg [238]. This is correlated with reduced proliferation rates within the cell columns, demonstrating that the rates of cell division are oxygen-dependent in both tumor and fetal cells.

Tumors are known to develop hypoxic areas within the tumor mass due to lack of sufficient blood supply to these sites [241,242]. At the same time, a reduced oxygen supply changes the glucose uptake of tumor cells [242] and increases the “evolutionary velocity”, thus raising the chances of new mutations [243]. Tumor sites where the pO_2_ level is too low develop necrosis. Interestingly, the tumor sites surrounding necrotic sites display pO_2_ levels in a range between 10 and 20 mmHg [241,242]. These are the sites of the highest proliferation rates [243], similar to what can be observed in the first trimester placenta. Glucose uptake follows, with higher rates of uptake at 10 mmHg compared to 20 mmHg [242]. From such sites, cells escape from the tumor and start to invade the surrounding tissues, which are normally vascularized and hence no longer show any signs of hypoxia.

### 2.7. Energy Metabolism in Tumor and Decidual Microenvironment

The low-oxygen microenvironment at the fetal–maternal interface in the first trimester of pregnancy is one of the major drivers of the glycolytic metabolic profile of the trophoblast (Figure 1). The fact that cytotrophoblasts are highly proliferative also supports their dependence on glycolysis, as this is a common feature of proliferating cells [244]. Glucose is the main carbohydrate catabolized by trophoblasts [245], and it is mostly provided by the rich supply from the endometrial glands [246]. Glucose import into the cells is governed by glucose transporters, with glucose transporter 1 (GLUT1) being predominant in the trophoblast of the first trimester placenta [247]. Other GLUTs, such as GLUT8 and GLUT9a, are present in the plasma membranes of the cells in the third trimester [248]. The high abundance of GLUTs, as well as their differential spatiotemporal distribution in the placenta, implies a regulated process that is mediated by transcriptional and epigenetic changes and initiated by nutritional, endocrine, and metabolic inputs from the microenvironment. Furthermore, GLUT expression and distribution can be distorted in pregnancy complications [248,249].

In many aspects, the metabolic phenotype of transformed tumor cells resembles the metabolic phenotype of highly proliferating cytotrophoblasts in the placenta. Proliferating tumor cells are largely considered glycolytic, meaning that they shift their metabolism from respiration (mitochondrial oxidative phosphorylation) towards glycolysis even in the presence of sufficient oxygen (also known as the Warburg shift) [250]. The glycolytic phenotype is common for dividing cells in general, because it allows for rapid glucose turnover and enables the quick production of building molecules for the rapidly proliferating cells [244]. This is achieved, for example, by shunting glycolysis intermediates to branching metabolic pathways, such as the pentose phosphate pathway for nucleotide production [251]. However, metabolic rewiring is an intricate part of cancer progression, and it is regulated by a delicate interplay between intrinsic factors (e.g., oncogenic mutations) and extrinsic factors from the TME [250,252,253,254]. The availability of nutrients within the unstable microenvironment influences the energy metabolism of cancer cells, which are able to shift between glycolysis and mitochondrial oxidative phosphorylation to produce ATP [250,255].

One more benefit coming from increased glycolysis that is utilized by both cancer and trophoblast cells is the reduction in reactive oxygen species (ROS), which can be destructive to cells with high oxidative phosphorylation [250]. In this sense, trophoblasts are more vulnerable to hyperoxia or fluctuating levels of oxygen than hypoxia [246]. In complications of pregnancy, such as early-onset pre-eclampsia or maternal diabetes, high levels of oxidative stress cause the release of factors that precipitate the maternal syndrome [246,256].

Finally, the increased nutrient uptake that can be attributed to both cancer and trophoblast cells can play a role in immune evasion. The results of cancer research have shown that cancer cells compete for nutrients with immune cells within the TME. For example, the increased uptake of glucose by cancer cells can deplete the TME of glucose, leading to dysfunctional T cells that are also highly glycolytic [257]. As a consequence of high glycolysis rates, lactate (the end product of aerobic glycolysis) is accumulated in both the tumor and decidual microenvironments [258]. Lactate can be used as an energy source by both the fetus and placenta, and it can also serve as a signaling molecule implicated in immune evasion [259]. In the TME, lactic acid has been shown to be a key signaling molecule in tumor cell migration, invasion, growth, angiogenesis, and immune escape [260,261].

Data from in vitro investigations of trophoblast metabolism are scarce, and their interpretation is often difficult. This is due to the use of non-physiological oxygen concentrations in in vitro cultures and a lack of good trophoblast-like cell lines or explant models [246,262,263]. Based on what we have learned so far, it seems plausible that the metabolism of both cytotrophoblasts and cancer cells is driven by the demand of their high proliferation rates. However, the high glycolysis rate does not change after implantation, even after circulation is established and oxygen levels are ‘normalized’ [258]. This dependence on glycolysis has provoked a novel hypothesis that proposes that the metabolic state of trophoblasts, rather than oxygen availability, regulates their fate, at least in part because the histone acetylation and open chromatin state of trophoblasts rely on the production of acetyl-CoA through glycolysis [264].

### 2.8. Long Non-Coding RNAs as Important Regulatory Players

Long non-coding RNAs (lncRNAs) are regulatory RNA molecules that are nowadays considered to be important players in numerous biological processes [265,266]. In human malignances, they have been implicated in cell proliferation, tumorigenesis, metastasis/invasion, immune evasion, and tumor metabolism, among others [265,266,267,268]. However, knowledge on expression profiles and functionality of lncRNAs in both tumor and trophoblast cells and/or their microenvironments is limited at the moment.

For example, the lncRNAs *MALAT1* and *LINC00473* have been implicated in regulating invasion in both settings. Experiments in a number of trophoblast-derived cell lines have shown that the lncRNA *LINC00473* appears to affect migration and invasion [269,270,271,272]; however, further experiments are needed to elucidate its role. In cancer, this lncRNA is upregulated in a variety of cancer types and has been associated with poor clinical outcomes [273,274,275]. Studies in cancer cell lines have also implicated *LINC00473* in the regulation of cell migration and invasion [274,275,276,277,278]. In trophoblast-derived cell lines, the silencing of *MALAT1* has been reported to inhibit migration and invasion [279,280,281,282]. In cancer, *MALAT1* has been suggested to promote invasion and metastasis; however, other reports have described a tumor-suppressing role of this lncRNA [283,284,285,286,287,288,289,290,291,292]. Another interesting example is the lncRNA *EPB41L4A-AS1*. Its low expression has been associated with poor prognosis in some cancer types [293,294]. The silencing of this lncRNA in cancer cell lines was found to trigger the Warburg effect, promoting aerobic glycolysis and glutamine metabolism [293]. As trophoblast and cancer cells both use glycolysis for fast growth, Zhu et al. investigated the regulatory effect of *EPB41L4A-AS1* on the metabolism of trophoblast-derived cell lines [295]. However, further investigation is required to explore the functional roles of these lncRNAs in trophoblasts and cancer cells. Furthermore, lncRNAs are being increasingly investigated for their regulatory roles within the TME [268,296,297,298,299,300,301]. Within the decidual microenvironment and the decidualization process, the regulatory involvement of these molecules is also being explored [302,303,304,305,306,307].

Further research is needed to investigate similarities and dissimilarities in expression, regulation, and functionality of lncRNAs between tumor and trophoblast cells and/or their microenvironments but could offer new perspectives for both fields of research. Notably, a number of lncRNAs are dysregulated in pregnancy pathologies [308,309,310].

## 3. Selected Pathologies of the Placenta and the Decidua

### 3.1. Preeclampsia and Intra-Uterine Growth Restriction

The identification of the etiologies of the two major pregnancy pathologies preeclampsia and intra-uterine growth restriction (IUGR) is still pending. Hence, the origins of both pathologies are still a matter of debate, and respective hypotheses are steadily developing [311,312,313]. Still, it is most likely that most cases of both pathologies are based on placental origins [21].

For preeclampsia, it is tempting to claim the dysregulation of the villous trophoblast as a cause [311]. This subtype of the trophoblast, serving as the epithelial cover of the placental villi, bathes in and continuously releases vesicles and factors into maternal blood [314]. The dysregulation of this layer during preeclampsia results in the release of so-far unknown factors that have negative impacts on the maternal vascular system. Depending on the susceptibility of the mother, she may develop the clinical symptoms of preeclampsia, hypertension, proteinuria, and other organ-based insults. This pregnancy pathology does not seem to be based on alterations of trophoblast invasion [21,311] but shows features of an upregulated inflammatory response of the mother to fetal components in her system.

In contrast, IUGR seems to develop following a failure in trophoblast invasion [311]. It has been shown that invasion of the uterine spiral arteries is reduced, leading to the reduced widening of the arterial ends connected to the placenta [315]. This in turn leads to a 10–20-fold increase in the flow velocity of maternal blood entering the placenta, which has dramatic morphological effects on its fragile villous tissue [315]. Villi are disrupted from the uterine wall, and the fetal vessels within the villous tissue experience a higher resistance. The combination of all these effects leads to an increase in placental oxygenation from less than 50 mmHg to more than 63 mmHg [316]. At the same time, the oxygen content in the umbilical vein, which transports blood from the placenta to the fetus, is reduced from 3.95 to 3.46 mmol/l [316]. Hence, while the placenta develops a hyperoxia, the fetus becomes hypoxic [240]. Higher oxygen levels in the placenta may further diminish the proliferation of trophoblasts serving as source for the invading population of trophoblasts, thus further decreasing the number of invading trophoblasts. Interestingly, so far, the initial trigger of reduced trophoblast invasion in IUGR is not known.

Both pregnancy pathologies, especially IUGR, may be linked to tumor biology. The changes in the maternal inflammatory response to so-far inert fetal/placental material could be used as a trigger in identifying respective molecular patterns in tumor cells that could be used for a tumor therapy. Knowledge on how the reduced invasion behavior of extravillous trophoblasts develops in IUGR may also be used to tone down the invasive capacity of tumor cells.

### 3.2. Placenta Accreta Spectrum

In the normal process of implantation, blastocysts attach to the decidua, which stimulates the proliferation and differentiation of the cytotrophoblast, eventually leading to the development of the two major subtypes of trophoblast: the villous and extravillous trophoblast [317]. Extravillous trophoblasts migrate through the decidua into the underlying inner third of the myometrium, thus forming the placental bed [317,318,319]. As they move through the maternal tissues, extravillous trophoblasts not only invade connective and muscular tissues but also penetrate into each and every luminal structure, including uterine arteries, veins, glands, and lymph vessels [15,17].

Placenta accreta spectrum disorders (PAS) fall into the group of placental implantation abnormalities [22]. Clinically, PAS features abnormal adherence of the placenta to the uterine wall and the absence of or/and a defective decidua [320]. Recently, the subgroups of placenta accreta, increta, and percreta were re-defined into grades 1, 2, and 3, respectively, according to the degree of invasiveness and the respective localization of the villous tissue. Histologically, in grade 1 (accreta), the placental villi directly attach to the myometrium, and in grade 2 (increta), placental villi intervene with the myometrial fibers or extend deep into the uterine vasculature. Grade 3 (percreta) features villous tissue/trophoblasts within or even penetrating the serosa; in severe cases, they also invade the bladder wall or other pelvic organs [320]. Extravillous trophoblasts are thought to undergo minimal or no EMT, which may influence the stringent regulation of their migratory capacity and invasiveness [321]. Their invasive driving forces steadily decrease during pregnancy [322], such that trophoblast invasion is usually restricted to the first half of gestation [323]. In PAS, extravillous trophoblasts seem to maintain their invasiveness into later stages of pregnancy and may also acquire EMT characteristics [324,325]. The activation of EMT has been proposed as a crucial dedifferentiation step from noninvasive to invasive phenotypes of epithelial cancer cells, cancer invasion, and metastasis [326,327].

When extravillous trophoblasts invade the decidua, they secrete regulatory factors such as proteolytic enzymes that dissolve ECM proteins. The secreted activators act on proteinases and MMPs present in the decidua. The intensive crosstalk between trophoblasts and cells within the decidua ensures controlled yet sufficient invasion [318]. Cells within the decidua secrete pro-invasive factors such as IL-1ß, IL-6. LIF, IL-11, IL-8, IL-15, IP-10, RANTES, eotaxin, and IL-7 and anti-invasive factors such as IL-10 and IL-12 [4,328]. IL-10 and IL-12 regulate MMP-2, MMP-9, and TIMP-1 expression [329]. MMP-2 and MMP-9 are the most studied MMPs secreted by extravillous trophoblasts [330]. The immunohistochemical analysis of MMP2 expression of placenta percreta tissue sections revealed higher expression of MMP-2, supporting the hypothesis of deeper invasion in such cases [331]. However, this could not be noted at the mRNA level in placenta accreta cases [332]. As placenta accreta cases show lower invasion severity compared to placenta percreta, the expression levels of MMP-2 probably differ between the grades of invasion. In cancer, higher MMP-2 and MMP-9 activities have been linked to not only tumor cell invasion but also angiogenesis [333,334,335,336,337].

The increased expression of VEGF, a pro-angiogenic factor, was observed in PAS [338]. Duzyj et al. suggested that the absence of endostatin, a VEGF-inhibiting factor expressed by decidual stromal cells, may promote trophoblast invasiveness [339,340]. Similarly, VEGF also plays a key role as a mediator of angiogenesis in cancer, where its expression is upregulated under hypoxic conditions [341]. The decidua, and to some extent the myometrium as well, regulate the extent and depth of trophoblast invasion through the secretion of TIMPs [342]. Specifically, TIMP-1 binds to complexes with MMP-9 and TIMP-2 regulates MMP-2 activity [343,344,345]. Higher numbers of extravillous trophoblasts were observed in the absence of decidua, supporting the hypothesis of the enhanced invasiveness of trophoblasts in the absence of decidua [346]. Similarly, in cancer, surrounding tissues secrete TIMPs, while excess TIMPs in the MMP–TIMP balance block the processing of tumor cell invasion [347,348,349]. Recently, an analysis of the immune cell population in PAS reveled decreased uNK cells, CD4+ T cells, Fox3P+ Tregs, and rare B cells with higher immune cell infiltration rates in the placental bed [350,351]. On the other hand, Schwede et al. reported increased Fox3P+ cell count and decreased counts of immature non-activated CD209+ DCs [352].

### 3.3. Choriocarcinoma

Choriocarcinoma is a rare and aggressive neoplasm originating from villous trophoblast, with varied incidence worldwide [353]. It can manifest as a gestational or non-gestational subtype and mainly occurs in women but can also occur in men as part of a mixed germ cell neoplasm. Choriocarcinoma develops from hyperplastic and anaplastic villous trophoblast, most frequently following a molar pregnancy [354]. The pathogenesis of choriocarcinoma is not fully understood. Studies have indicated that villous cytotrophoblasts undergo malignant transformation and thus do no longer only differentiate into the syncytiotrophoblast, but they may also upregulate EMT feature and thus differentiate into invasive and malignant trophoblasts [355,356].

Since choriocarcinoma is a rare neoplasm [354], only a few studies have focused on the investigation of the TME with limited numbers of tumor samples [357,358,359]. All of these studies showed that the immune cell infiltration occurred locally or was even missing within the tumor, whereas the vigorous infiltration of NK cells, cytotoxic T cells, and Tregs was detected in the adjacent tissue [357,358,359]. Furthermore, it has been shown that malignant trophoblasts and the villous syncytiotrophoblast express the immune inhibitory PD-L1 ligand [357]). However, data on that topic are rather preliminary, and further comprehensive studies are needed to fully elucidate the development and TME of choriocarcinoma.

## 4. Conclusions

Trophoblast and tumor cells exhibit many striking similarities [7,8], and they are both supported by an abetting microenvironment [9]. Interestingly, similarities in gene expression and DNA methylation have also been highlighted [8,89,184,360,361,362,363,364,365,366]. In this article, we discuss the similarities and dissimilarities in the regulatory processes driving trophoblast and tumor cell fate, with a focus on the role of the decidual and tumor microenvironments (summarized in Table 1). In pregnancy, decidual cytotoxic T cells have the potential to recognize the semi-allogeneic fetal cells through fetal antigens [158], and, to ensure a healthy pregnancy, these must be tolerated by the maternal immune cells in the decidual microenvironment. In cancer, cytotoxic T cells can recognize tumor cells via tumor antigens, which comprise a number of categories, including neoantigens, HERV-derived antigens, cancer testis antigens, and tumor-associated antigens [172,367,368,369]. Hence, tumor cells are pressured to find mechanisms to circumvent their elimination by immune cells in the TME [172]. Particularly, the interactions of immune inhibitory ligands with the corresponding co-inhibitory receptors, which suppress immune cell responses and induce suppressive immune cell types, are involved in establishing these supporting microenvironments [182,184,192,370]. Interestingly, non-classical MHC class I molecules are emerging as potent players in immunomodulatory processes, particularly involving NK cells [151,370]. Both microenvironments support the promotion of invasion and angiogenesis, as outlined in the designated sections of this review. As the developing fetus and a growing tumor increase in size, they need to reinforce their supply with oxygen and nutrients. To do so, trophoblasts and tumor cells alter their microenvironment by releasing a plethora of matrix-degrading proteins [42,335] and pro-angiogenic signals [112,117]. These signals activate and attract endothelial cells on the one hand and recruit leukocytes on the other hand [126,127,136,137], both of which further participate in tissue remodeling processes and angiogenesis.

Nevertheless, we recommend caution in defining the placenta as a well-behaved tumor, as this term may be misleading. While cancer cells use similar pathways and mechanisms, there are crucial differences in regulatory processes between trophoblast and tumor cells, as well as between their microenvironments. Importantly, trophoblast cells only proliferate or invade in a tightly controlled fashion [1,8,10]. The proliferative capability is limited to trophoblasts residing on the basement membrane of placental tissues, and the invading extravillous trophoblasts do no longer proliferate [1]. In contrast, cancer cells exhibit uncontrolled proliferation and show plasticity in transitioning between invasive and proliferative states, which has been suggested to be regulated by microenvironmental conditions [371,372,373]. Moreover, while a high proliferation rate is essential for the rapid growth of embryonic and placental tissues in early stages of pregnancy, transition to cellular differentiation and senescence occurs towards term. The disruption of this balanced regulation could lead to gestational trophoblastic diseases [88]. Tumor-suppressor genes may play a key role in regulating trophoblast cell expansion and invasion processes. However, these are tightly regulated, whereas in many cancers, such genes are commonly mutated, which can result in tumor formation or growth [52]. Tumor suppressors (wild type and mutated) are subject to a complex array of post-translational modifications that may differentially affect stability, high molecular complex formation, and activity of tumor suppressors in trophoblasts and tumor cells. Additionally, uNK cells are highly abundant in the decidual microenvironment and are thought to have important regulatory roles in pregnancy, including regulating invasion and spiral artery remodeling [33,34]. In the tumor setting, NK cells play an important part in immune surveillance, but tumor cells can develop a range of evading strategies, which include limited NK cell infiltration into solid tumors, NK cell dysfunction [172,185,232,233], and possibly even the adoption of a uNK cell-like phenotype [173,174,175,176,177,178,179]. Furthermore, even though both cell types show similar metabolic states that are strongly reliant on glycolysis, cancer cells also demonstrate astonishing metabolic flexibility [264], while trophoblasts maintain their metabolic state [374]. Additionally, alterations in placental energy metabolism can be the underlying factors of placental pathologies, such as preeclampsia [374].

Taken together, although trophoblast and tumor cells share an astonishing degree of similarity, including an abetting microenvironment, both cell types are clearly distinguishable. Trophoblasts are tightly controlled via intrinsic and extrinsic factors, while tumor cells can escape many control mechanisms.

## Figures and Tables

**Table 1:** An abetting microenvironment sustains both trophoblasts and tumor cells, as well as influences/regulates invasion, angiogenesis, and immune tolerance/evasion, among others. In comparison to tumor cells, the metabolic, proliferative, migrative, and invasive states of trophoblast cells are under tight regulatory control. This table summarizes the main characteristics/aspects discussed in this review.

Topic	Tumor Setting	Decidual Setting
Growth Suppression in Tumor and Decidual Microenvironment	Tumor-suppressor genes (e.g., *Rb*, *PTEN,* and *p53*) are mutated in many cancers Post-translational modifications of tumor suppressors regulate their function Many different binding partners of tumor suppressors	Tumor-suppressor genes not mutated in trophoblasts Post-translational modifications of tumor suppressors regulate their function Many different binding partners of tumor suppressors
Proliferative Signaling in Tumor and Decidual Microenvironment	Dysregulated proliferation (through growth pathway-activating oncogenes) Sustained proliferation refractory to growth factors in the microenvironment Transition between invasive and proliferative states	Tightly regulated proliferation Proliferation regulated by growth factors in the microenvironment (e.g., EGF, HGF, IGF, and PIGF) Resident placental trophoblasts proliferate Invading extravillous trophoblasts do not proliferate
Angiogenesis in Tumor and Decidual Microenvironment	Increasing demand for oxygen and nutrients Release of pro-angiogenic factors Recruitment of leukocytes (macrophages, NK cells, and DCs) Vasculogenic mimicry	Increasing demand for oxygen and nutrients Release of pro-angiogenic factors Recruitment of leukocytes (macrophages, uNK cells, and DCs) Spiral artery remodeling
Evasion of Immune Destruction in Tumor and Decidual Microenvironment	TME contains NK cells, macrophages, DCs, neutrophils, T cells, Tregs, and B cells Immune suppression facilitated by co-inhibitory signals, secreted immunosuppressive cytokines, metabolites (e.g., IDO), non-classical MHC class I molecules, and/or the recruitment of Tregs and/or NK cells	Decidua contains uNK cells, macrophages, DCs, T cells, and Tregs Immune tolerance facilitated by co-inhibitory signals, secreted immunosuppressive cytokines, metabolites (e.g., IDO), non-classical MHC class I molecules, and/or recruitment of Tregs and/or uNK cells
Promotion of Invasion in Tumor and Decidual Microenvironment	Changes in cellular programs e.g., responsible for the loss of cell–to-cell contacts Uncontrolled invasion of the surrounding tissues with no endpoint (forming distant metastases) Dysregulated HOXA10 pathway Hormonal influence on invasion depending on tumor type Cells of the TME are involved in regulating invasion	Changes in cellular programs e.g., responsible for the loss of cell–to-cell contacts Highly organized differentiation pattern with invasion endpoint (decidua plays important role) Regulated HOXA10 pathway Hormonal regulation of invasion Cells of the decidual microenvironment are involved in regulating invasion
Chemo-Physical Aspects of Tumor and Decidual Microenvironment: Oxygen as an Example	Proliferation at various oxygen levels Transition between invasive and proliferative states Invasion towards higher oxygen levels	Proliferation only at low oxygen levels Separation of proliferation and invasion Invasion towards higher oxygen levels
Energy Metabolism in Tumor and Decidual Microenvironment	Glycolytic Glucose provided by intra-tumoral vasculature (variable supply) Lactate as an energy source and signaling molecule Metabolic flexibility and plasticity Rewired metabolism promotes tumor initiation, growth, and metastasis	Glycolytic Glucose provided from endometrial glands (steady supply) Lactate as an energy source and signaling molecule Regulated metabolic state Altered energy metabolism in placental pathologies (e.g., preeclampsia)
Long Non-Coding RNAs as Important Regulatory Players	Regulatory players in many biological processes including invasion A number of lncRNAs are dysregulated in malignances	Regulatory players in many biological processes including invasion A number of lncRNAs are dysregulated in pregnancy pathologies

## Data Availability

Not applicable.
